# Efficacy of ambulance air purifiers with different photocatalytic oxidation components in the removal of *Bacillus subtilis* spores

**DOI:** 10.1038/s41598-026-36581-4

**Published:** 2026-01-17

**Authors:** Akkrapol Poohpajit, Santisith Khiewkhern, Chuleewan Thunyasirinon, Prapat Pentamwa

**Affiliations:** 1 Faculty of Public Health, Mahasarakham University, Maha Sarakham, 44150 Thailand; 2 Institute of Public Health, Suranaree University of Technology, Nakhon Ratchasima, 30000 Thailand

**Keywords:** Photocatalytic oxidation, UVA/TiO₂, Ambulance disinfection, Airborne disinfection, Surface disinfection, *B. subtilis* spores, Biotechnology, Environmental sciences, Materials science, Microbiology

## Abstract

Ambulances are enclosed environments that carry a high risk of airborne and surface microbial transmission, yet effective disinfection technologies remain limited. This study evaluated four photocatalytic oxidation (PCO) configurations—O₃+UVA + TiO₂, UVA + TiO₂, O₃+UVC + ZnO, and UVC + ZnO—against *B. subtilis* spores. The testing employed a prototype air purification system for ambulance applications, where the photocatalyst TiO_2_ or ZnO was integrated into a filter medium. This system operated in combination with its corresponding UV light source (UVA or UVC) and an optional ozone generator; all housed within a laboratory-simulated ambulance cabin. (8.998 m³), where spores at 1.5 × 10⁸ CFU/mL (8 mL) were spray misted using a nebulizer and sampled using an Andersen Impactor, following the NIOSH method. Disinfection efficacy was quantified as the percentage reduction of *B. subtilis* spores in the air and on surfaces. Among the tested systems, efficacy ranked as UVA + TiO₂ > O₃+UVA + TiO₂ > O₃+UVC + ZnO > UVC + ZnO. UVA + TIO_2_ achieved the most rapid and stable disinfection among the tested systems under controlled conditions, reducing airborne spores by > 80% within 15 min, achieving complete removal within 90 min, and reducing surface contamination by 96.77% at 120 min. In contrast, ZnO- and UVC-based systems exhibited lower or inconsistent performance. These findings identify UVA + TiO₂ photocatalysis as a safe, ozone-free, and highly effective strategy for ambulance air purification. Its rapid and durable antimicrobial action demonstrates clear advantages over approaches based on ozone or UVC, offering practical benefits for infection control in emergency medical services and providing a foundation for further optimization of photocatalytic technologies in healthcare settings.

## Introduction

The ongoing transmission of respiratory infectious diseases remains a critical global public health challenge, adversely affecting health outcomes, increasing the risk of mortality and disability, extending hospital stays, and driving up healthcare costs^1^. Several infectious diseases are highly severe, particularly emerging infections such as severe acute respiratory syndrome (SARS), Middle East respiratory syndrome coronavirus (MERS-CoV), and the novel coronavirus disease 2019 (COVID-19), as well as re-emerging diseases like pulmonary tuberculosis (TB) in its infectious stage, which continue to pose a significant disease burden in many countries^2^. While these infections often manifest as severe respiratory illnesses, the resulting pathogens can be transmitted through multiple routes. Airborne transmission becomes particularly significant when patients cough or sneeze. These actions generate fine aerosol droplets containing pathogens, which not only remain suspended in the air but also readily deposit on surrounding surfaces, including medical equipment, floors, walls, and ceilings.

In poorly ventilated environments, the risk of exposure to respiratory infectious diseases is elevated for individuals present, especially in healthcare settings with high patient density^3^. In hospital environments with a high risk of airborne pathogen contamination, particularly in areas with continuous movement of patients and healthcare personnel, indoor air quality represents a critical determinant of health outcomes. Compromised air quality in such settings can adversely affect patients, healthcare workers, and visitors, thereby increasing the risk of infection transmission and highlighting the necessity of effective air disinfection strategies^4,5^. Numerous studies have highlighted infection prevention and safety measures in hospital environments^6–8^. However, the quality of air inside ambulance environments is associated with a particularly high risk of infection transmission and has often been overlooked, despite the frequent detection of multidrug-resistant organisms (MDROs), including methicillin-resistant *Staphylococcus aureus* (MRSA), vancomycin-resistant *Enterococcus* (VRE), and extended-spectrum β-lactamase (ESBL)-producing bacteria^9,10^. These pathogens have the potential to cause severe infections and cross-border outbreaks, posing significant threats to global health security^11^. These factors can adversely affect treatment outcomes and contribute to severe illnesses globally. The World Health Organization (WHO) has recognized respiratory infectious diseases and antimicrobial resistance (AMR) as among the top ten global health threats^12,13^.

**Fig. 1:**
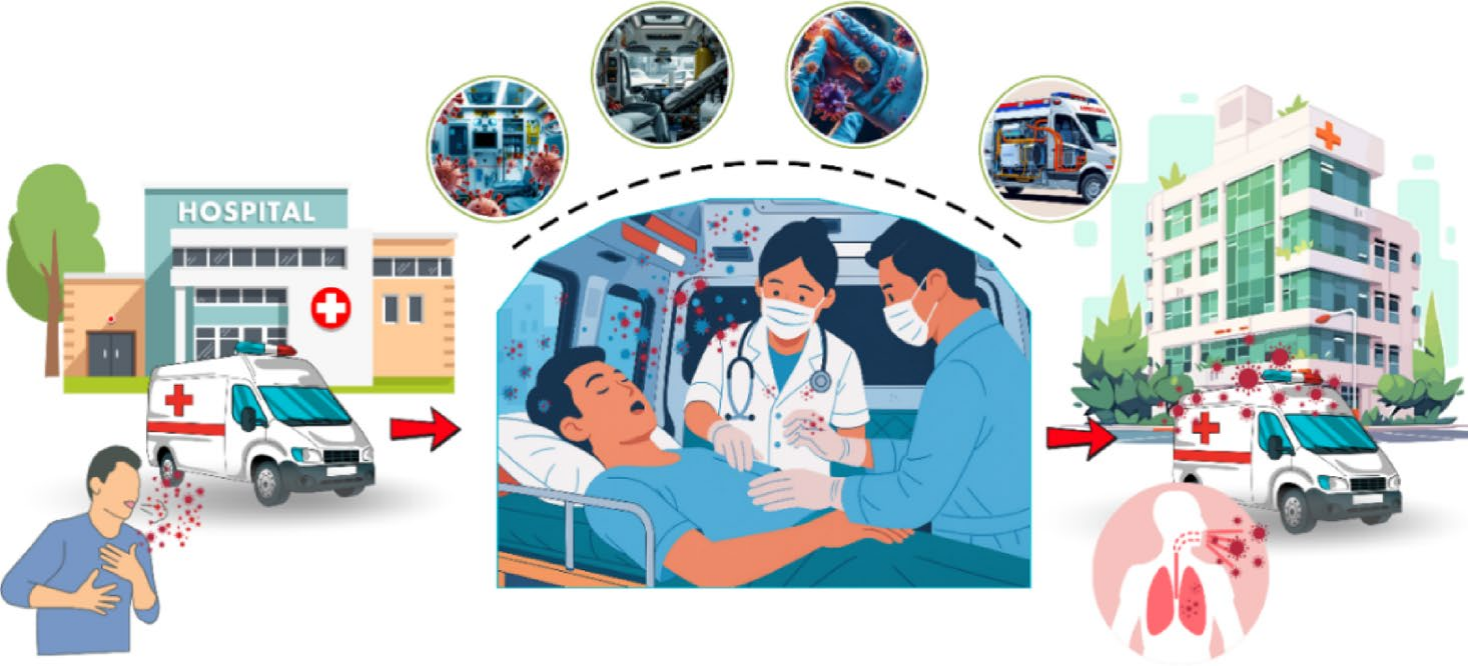
Sources and transmission pathways of microorganisms in ambulances.

Ambulances, which function as mobile emergency medical services (EMS), play a critical role in the emergency care system. As enclosed environments shared by healthcare personnel, patients, and relatives, both air and surface cleanliness are essential. This is particularly crucial when transporting patients with severe respiratory infections, where pathogens can readily spread to healthcare workers and other occupants. The spread of these pathogens is often facilitated by droplets, ranging from 1 to 100 microns in size, generated by coughing or sneezing^14^. which may cause airborne microorganisms to settle on various surfaces within the ambulance^15^. Several studies have reported contamination of ambulances by pathogenic microorganisms, including *Staphylococcus* spp., MRSA, *Klebsiella pneumoniae*, *Escherichia coli*, VRE, and other drug-resistant pathogens^16^. The most frequent sites of accumulation for these pathogenic microorganisms were identified as the oxygen tanks, ambulance floors, and rear grab handles^17^. Due to the spatial limitations of ambulances, which are typically converted from standard vans, ventilation is often inefficient. As a result, the internal air circulates in a closed loop, allowing microorganisms to accumulate and persist for extended periods^18^. The microbial sources and transmission dynamics within ambulance environments—including airborne dissemination and surface deposition—are illustrated in Fig. 1, underscoring the heightened infection risks inherent in confined prehospital settings. Although guidelines exist for cleaning ambulances after patient transport, such as ventilating the vehicle by keeping the doors open for 30 min, adherence to these practices is often inconsistent, limiting their effectiveness in reducing microbial contamination^19^. As such, a concerning rate of occupational exposure related illnesses among ambulance personnel continues to be observed, averaging 1.2 cases per 1,000 workers^20^. The spread of multidrug-resistant and airborne pathogens in emergency medical services poses a significant threat to global health security. These infections among healthcare personnel directly impact their capacity to respond to global health emergencies, compromising disease control, affecting medical staff, and jeopardizing the continuity of healthcare systems worldwide.

Disinfection of microorganisms in ambulances is crucial for reducing the risk of cross-contamination among patients, healthcare personnel, and staff. Given that pathogens can be transmitted through the air or contaminate various surfaces, efforts have been made to develop disinfection technologies specifically for ambulances. Ultraviolet C (UV-C) irradiation, in particular, has been reported to effectively reduce the concentration of airborne pathogens^21^. Furthermore, some studies have explored the synergistic use of UV irradiation with chemical disinfectants to enhance overall efficacy^22^. However, UV-C irradiation has significant limitations. Its efficacy is reduced by obstructions, and it poses health risks, including keratitis from even brief exposure, and skin irritation or inflammation^23^. Furthermore, the combined use of chemical disinfectants with UV-C irradiation can generate chemical aerosols, which may adversely affect medical equipment within the ambulance^24^. Given the limitations of using UV-C irradiation in combination with chemical disinfectants, new approaches, such as Photocatalytic Oxidation (PCO) technology, have emerged. This technology utilizes light energy to activate a photocatalyst^25^. PCO technology, in the form of an air purifier, efficiently oxidizes pathogens by producing reactive oxygen species (ROS), such as hydroxyl radicals (•OH) and superoxide (O₂-•). These species effectively inactivate a wide range of microorganisms, including fungi, bacteria, viruses, and drug-resistant pathogens^26^. This study aimed to compare the efficacy of four distinct PCO air purifier systems— UVA + TiO_2_, O_3_ + UVA + TiO_2_, UVC + ZnO and O_3_ + UVC + ZnO—in the removal of *B. subtilis* spores within a simulated ambulance cabin. The UVC + TiO_2_ system was deliberately excluded due to material science limitations, specifically TiO_2_’s poor UVC absorption and rapid photocorrosion^27,28^ which compromises its practical stability. The findings not only offer practical guidance but also provide a foundation for implementing this technology to address both airborne and surface contamination in ambulances, a high-risk environment where the critical issue of respiratory pathogen transmission is currently underserved by appropriate technological solutions.

## Experimental method

This experimental study aimed to compare the efficacy of ambulance air purifiers with different PCO components. The efficacy was measured by the removal of *B. subtilis* spores in both airborne and surface-bound forms.

### Ambulance air purifiers

**Fig. 2:**
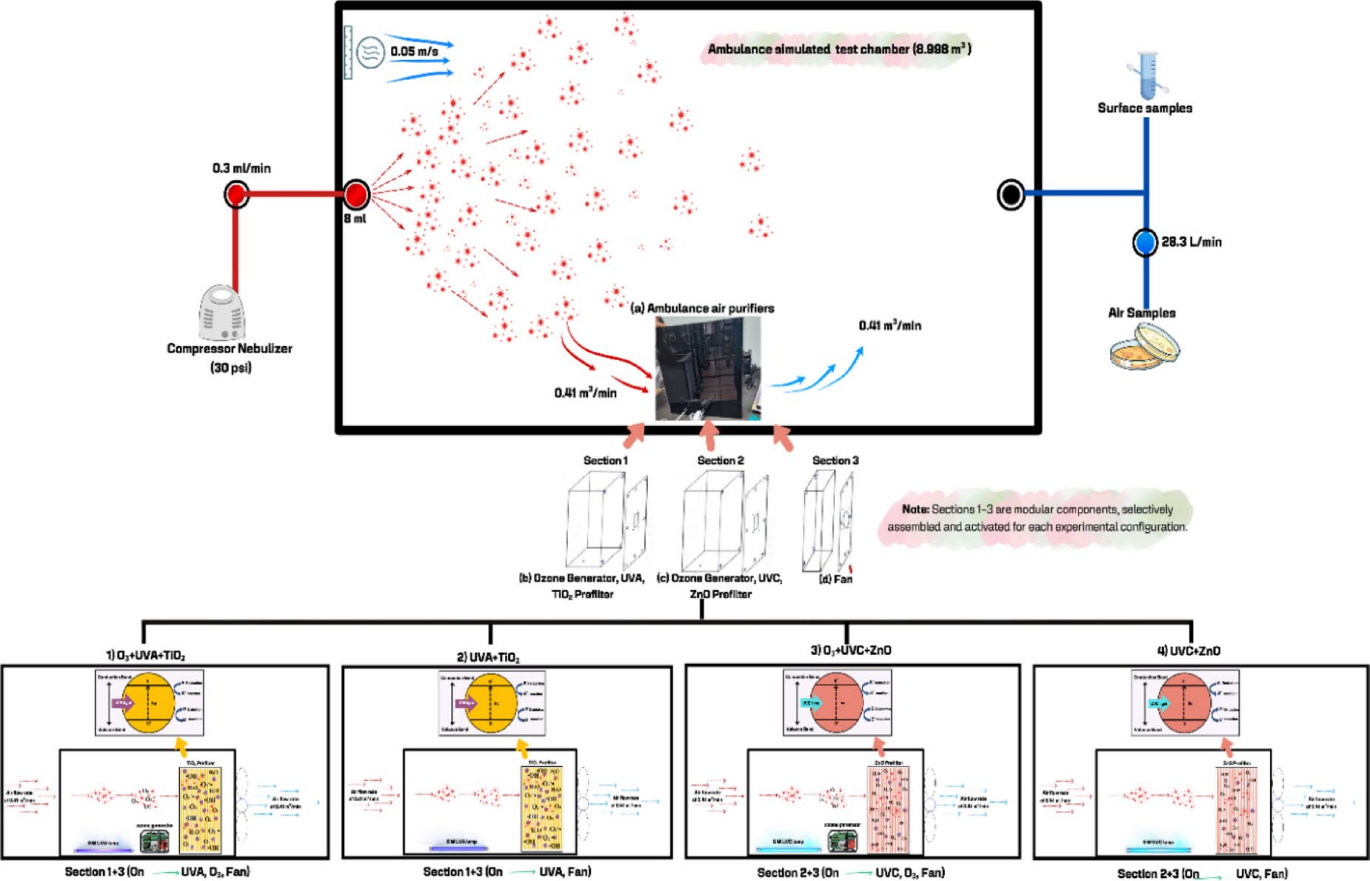
Schematic of the ambulance-simulated test chamber and the modular photocatalytic air purifier.

The prototype device was engineered from three independent modular sections, each equipped with its own dedicated on–off switch. This modularity allowed for the selective assembly and activation of components to create various photocatalytic configurations for comparative testing. Section 1 (20 × 40 × 40 cm) contained a 6 W UVA lamp and a 20 × 20 cm TiO_2_-coated prefilter, and an optional ozone generator. Section 2 (30 × 40 × 40 cm) incorporated a 6 W UVC lamp, a 20 × 20 cm ZnO-coated prefilter, and an optional ozone generator. Section 3 (10 × 40 × 40 cm) housed a 120-mm fan, which provided the necessary airflow through the system in Fig. 2.

Because these units operated independently, the sections could be physically and electronically combined in different arrangements (e.g., Sect. 1 + 3 or 2 + 3) to achieve the four PCO configurations evaluated in this study. The internal fan delivered a measured airflow rate of 0.41m^3^/min, achieving an Air Change Rate (ACH) of 12 h^− 1^ within the 8.998 m^3^ test chamber. Both UVA and UVC lamps provided 6 W output. Light intensities measured directly on the TiO_2_/ZnO catalyst surfaces were 5.9 µW/cm^2^ (UVA) and 3.2 µW/cm^2^ (UVC), as determined using a calibrated radiometer.

### Coating of prefilters with metal oxide nanoparticles

Prefilter sheets (20 × 20 cm) were coated for use as components in an ambulance air purification device. Two types of metal oxide nanoparticles, TiO₂, and ZnO, were deposited on the sheets using the sol-gel method^29^, as described below.

For the TiO₂ coating, a 1000 mL beaker was filled with 500 mL of deionized water and placed on a magnetic stirrer. A magnetic stir bar was added, and the solution was stirred at 100 rpm. Polyethylene glycol (PEG) was then added as a binder at 0.3% of the weight of the TiO₂ powder^30,31^. The prefilter sheets were prepared by sequentially washing with acetone, followed by ethanol, and finally rinsed with deionized water. The sheets were dried in an oven at 103–105 °C until completely dry. Each sheet was then immersed in the TiO₂ suspension for 1 min and subsequently dried in an oven at 103–105 °C for 1 h. This coating procedure was repeated five times^32^.

To prepare the ZnO coating, 2 g of zinc acetate dihydrate and 8 g of sodium hydroxide (NaOH) were weighed out. The zinc acetate dihydrate was dissolved in 15 mL of deionized water, and the NaOH was dissolved in 10 mL of deionized water in a fume hood^33^. Each solution was stirred with a magnetic stir bar at 100 rpm for 5 min. The NaOH solution was then added dropwise to the zinc acetate solution, and the mixture was stirred at 100 rpm for an additional 5 min. Ethanol was slowly added from a burette until a white precipitate formed^34^. Subsequently, 5 mL of PEG was dissolved in deionized water and added to the precipitate suspension. The final suspension was stirred at 100 rpm for 5 min to obtain the ZnO suspension. The prefilter sheets were prepared by sequentially washing with acetone, followed by ethanol, and finally rinsed with deionized water. The sheets were then dried in an oven at 103–105 °C until they were completely dry. Each sheet was immersed in the ZnO suspension for 1 min and then dried in an oven at 103–105 °C for 1 h. This dipping and drying procedure was repeated a total of five times^34^.

### Preparation of *B. subtilis* spores

*B. subtilis* spores were purchased from the Thailand Institute of Scientific and Technological Research in dry powder form. The powder was dissolved in sterile deionized water under aseptic conditions. After thorough mixing to obtain a homogeneous microbial suspension, the suspension was pipetted onto Trypticase Soy Agar (TSA) plates and incubated at 37 °C for 7 days^35^. Once colonies of *B. subtilis* had formed, spores were prepared by diluting the culture in deionized water and heating in a water bath at 80 °C for 10 minutes^36^ to eliminate vegetative cells, leaving only spores. The suspension was then centrifuged at 2,000 rpm for 5 minutes^37^, and the resulting spores were resuspended in 30 mL of sterile 0.85% (w/v) sodium chloride (NaCl) solution. A 10 mL aliquot of this suspension was used to measure turbidity with a densitometer (DEN-1B) at a wavelength of 550 nm^38^. The spore suspension was adjusted to a concentration of 1.5 × 10⁸ CFU/mL, corresponding to a 0.5 McFarland standard.

### Ambulance simulated test chamber and setup

**Fig. 3:**
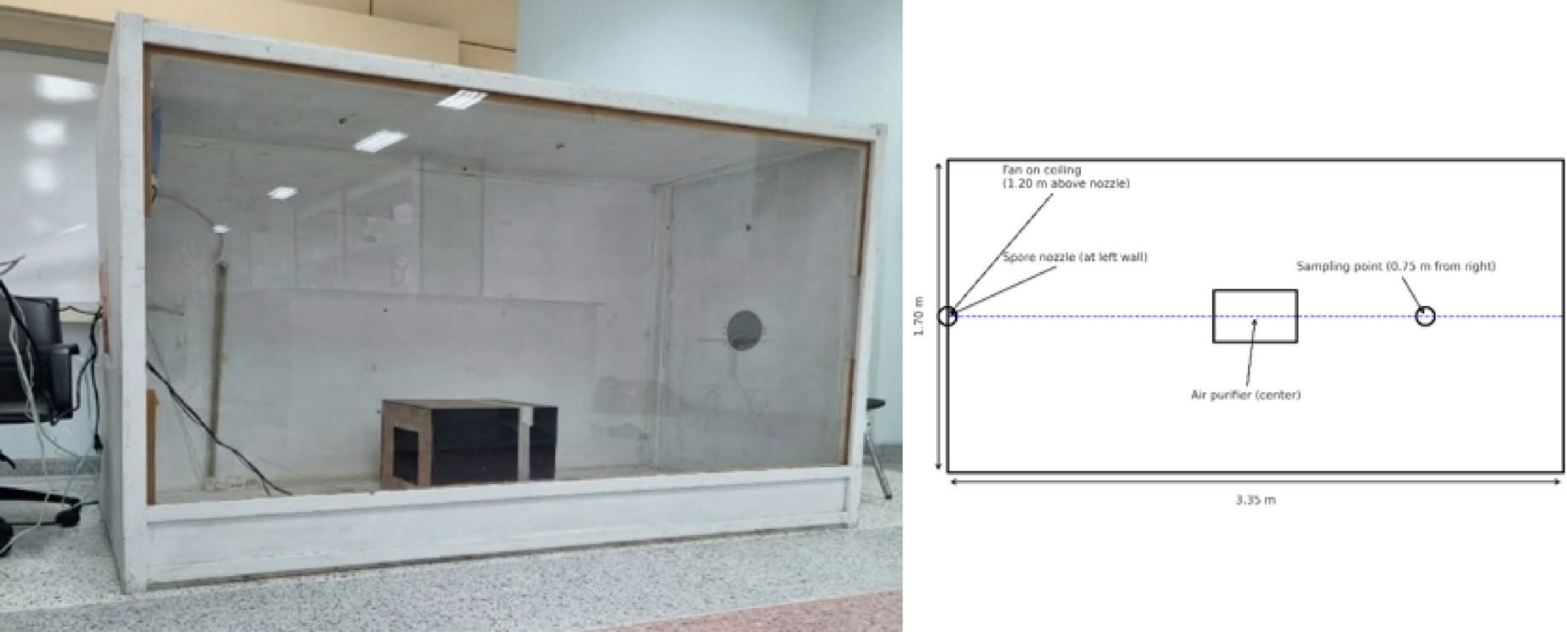
Simulated ambulance test chamber and setup.

The chamber was constructed with a volume of 8.998 m^3^ to accurately replicate the patient compartment volume of a standard ambulance, as illustrated in Fig. 3. To ensure representative airflow conditions, the fan used for air circulation was strategically located on the ceiling to simulate the exact position of the air outlet from the ambulance’s air conditioning system. This fan was operated at a constant outlet airflow velocity of 0.05 m/s, a value specifically chosen to achieve an air change rate ACH of approximately 12 h^− 1^ a rate often recommended by health authorities (e.g., ASHRAE/CDC) for controlling airborne contamination in high-risk, confined healthcare settings (such as emergency transport vehicles or isolation rooms).

Conversely, the air purifier unit was centrally positioned solely to facilitate a standardized comparison of the intrinsic PCO disinfection efficacy. We acknowledge that the chamber’s structure was simplified for standardized laboratory testing and was not intended to perfectly match the complex PVC or Vinyl compositions commonly found in real ambulances. Nevertheless, this controlled setup was critical as it allowed us to isolate and precisely measure the intrinsic PCO disinfection performance, while successfully maintaining a representative air circulation pattern and meeting hygienic ACH standards.

### *B. subtilis* spore removal test

The experiment was initiated using a nebulizer (Omron Compressor Nebulizer NE-C101) to aerosolize *B. subtilis* spores at a concentration of 1.5 × 10⁸ CFU/mL, with a total volume of 8 mL^39^, into the laboratory-simulated ambulance cabin. During aerosolization, the internal fan of the chamber operated for 30 min to ensure uniform spore distribution while the air purification devices remained off. Air samples were collected at 0, 15, and 30 min (baseline spore concentrations) using a single-stage impactor (SKC, Inc., model Biostage) at a flow rate of 28.3 L/min for 3 min per sample, following NIOSH Method 0800^40^. The collected samples were plated on 90 mm. Trypticase Soy Agar (TSA) plates. Surface samples were collected using sterile swabs moistened with 0.85% (w/v) sodium chloride solution. Defined 10 × 10 cm. areas were swabbed with the swab held at approximately a 30° angle to the surface. Surface samples were collected at 30 min (baseline) before the activation of the air purification devices.

For each test, an air purification component (O₃+UVA + TiO₂, UVA + TiO₂, O₃+UVC + ZnO, or UVC + ZnO) was operated after 30 min of aerosolization, with air samples collected at 45, 60, 75, 90, 105, 120, 135 and 150 min, and surface samples collected at 90 and 150 min. Each experiment was replicated three times. Surface sample tubes were processed by transferring 1 mL of swab suspension into 9 mL of 0.85% (w/v) NaCl solution and mixing thoroughly. A 0.1 mL aliquot was then spread onto TSA plates, which were incubated at 37 °C for 7 days. Air samples were processed in the same manner.

All experiments were conducted under Biosafety Level 1 (BSL-1) conditions. Due to aerosol generation, decontamination and infection control procedures were strictly followed after each experiment, in accordance with hospital infection control standards, including continuous UV-C exposure for 4–6 hours^41^. The absence of *B. subtilis* contamination was confirmed before repeating the experiment.

## Evaluation and data analysis

The performance of the air purification devices in removing *B. subtilis* spores was evaluated by calculating the percentage reduction of spores over time. Spore removal percentage (%) at each sampling time was determined using the following formula:

Removal (%) = ((C₀ − Ct) / C₀) × 100 where *C*_*0*_ represents the initial spore concentration, and *C*_*t*​_ is the spore concentration at time.

The removal efficiencies of the air purification devices with different photocatalytic oxidation configurations were compared. The results were presented as graphs showing the percentage of *B.subtilis* spores inactivated over time, allowing a direct comparison of the effectiveness of each PCO configuration in reducing both airborne and surface-bound spores.

## Results and discussion

### Characterization of prefilter physical properties

The elemental composition of the prefilter sheets was analyzed using Energy Dispersive X-ray (EDX) spectroscopy, with the results summarized in Table 1. For the prefilter sheets coated with TiO₂, titanium (Ti) was the predominant element, accounting for 51.99% of the composition. In the sheets coated with ZnO, zinc (Zn) was detected at 29.82% while carbon (C) was the most abundant element, followed by oxygen (O). Both titanium^42^ and zinc^43^ are stable elements that function effectively as catalysts in photocatalytic reactions.

**Table 1:** Types and amounts of elemental components detected in prefilter sheets (%).

Spectrum	C	O	Na	Cl	Zn	Ti
Control	69.48	25.31	2.48	2.73	-	-
TiO_2_	32.92	9.93	3.14	2.14	-	51.99
ZnO	36.23	31.31	2.11	1.08	29.82	-

**Fig. 4:**
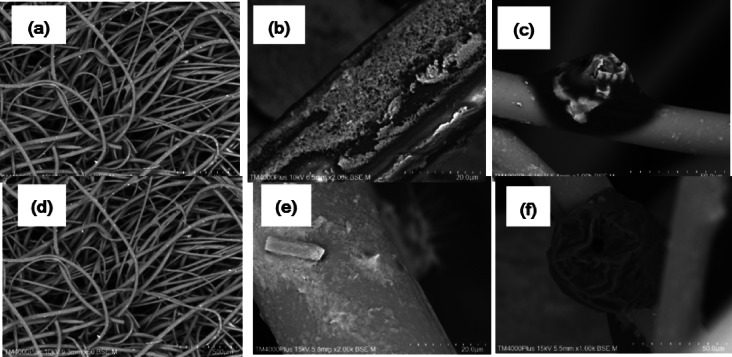
SEM images showing the surface morphology of prefilter samples: (**a**) uncoated prefilter (60 BSE M), (**b**) TiO₂-coated prefilter (2.00k BSE M), (**c**) disinfection filter (1.00k BSE M), (**d**) uncoated prefilter (60 BSE M), (**e**) ZnO-coated prefilter (2.00k BSE M), and (**f**) disinfection filter (1.00k BSE M).

The surface morphology of the nanoparticle-coated prefilter sheets was examined using scanning electron microscopy (SEM). The analysis revealed that the prefilter surfaces were irregular and contained pores of various sizes distributed across the sheets^44^, as shown in Fig. 4. These pores can facilitate microbial adhesion^45^, which is a significant factor in the photocatalytic oxidation process. Upon UV light application, this process effectively removed *B.subtilis* spores.

### Removal of *B. subtilis* spores in air

**Fig. 5:**
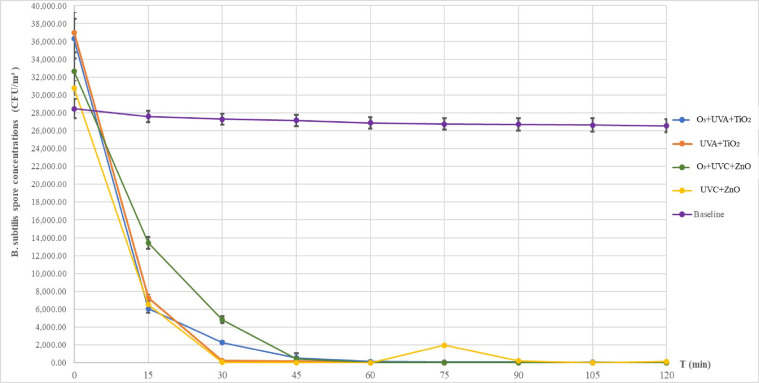
Airborne concentrations of *B. subtilis* spores over time following treatment with different PCO configurations.

Figure 5 illustrates the time-dependent reduction of airborne *B. subtilis* spores for each PCO configuration, with error bars representing the experimental variability. The O₃+UVA + TiO₂ configuration demonstrated a rapid reduction in the initial spore concentration, from 36 *±* 8 × 10^3^ CFU/m^3^ to 2.5 *±* 1.1 × 10^3^ CFU/m^3^ (83.29% reduction) within 15 min. It continued to remove spores, achieving an overall efficiency of 99.97% with only 12 *±* 21 CFU/m³ remaining after 120 min. Similarly, the UVA + TiO₂ configuration showed rapid and consistent spore removal, decreasing the initial concentration from 37.0 *±* 6.4 × 10^3^ CFU/m^3^ to 7.2 *±* 0.4 × 10^3^ CFU/m^3^ (80.43% reduction) within 15 min. This configuration achieved complete spore elimination (100% removal) from 90 min onward. In contrast, the O₃+UVC + ZnO configuration exhibited a slow initial spore reduction, with concentrations decreasing from 32.7 *±* 1.9 × 10^3^ CFU/m^3^ to 14.8 *±* 5.5 × 10^3^ CFU/m^3^ (58.91% reduction) within 15 min. Although its performance improved in the later stages, the early-stage removal rate was markedly lower than that of the other configurations. Finally, the UVC + ZnO configuration showed high initial removal, with spore concentrations dropping from 30.8 *±* 16.4 × 10^3^ CFU/m^3^ to 6.5 *±* 0.4 × 10^3^ CFU/m^3^ within 15 min. However, temporary increases in spore count were observed at 75 and 120 min, indicating microbial recovery.

**Fig. 6:**
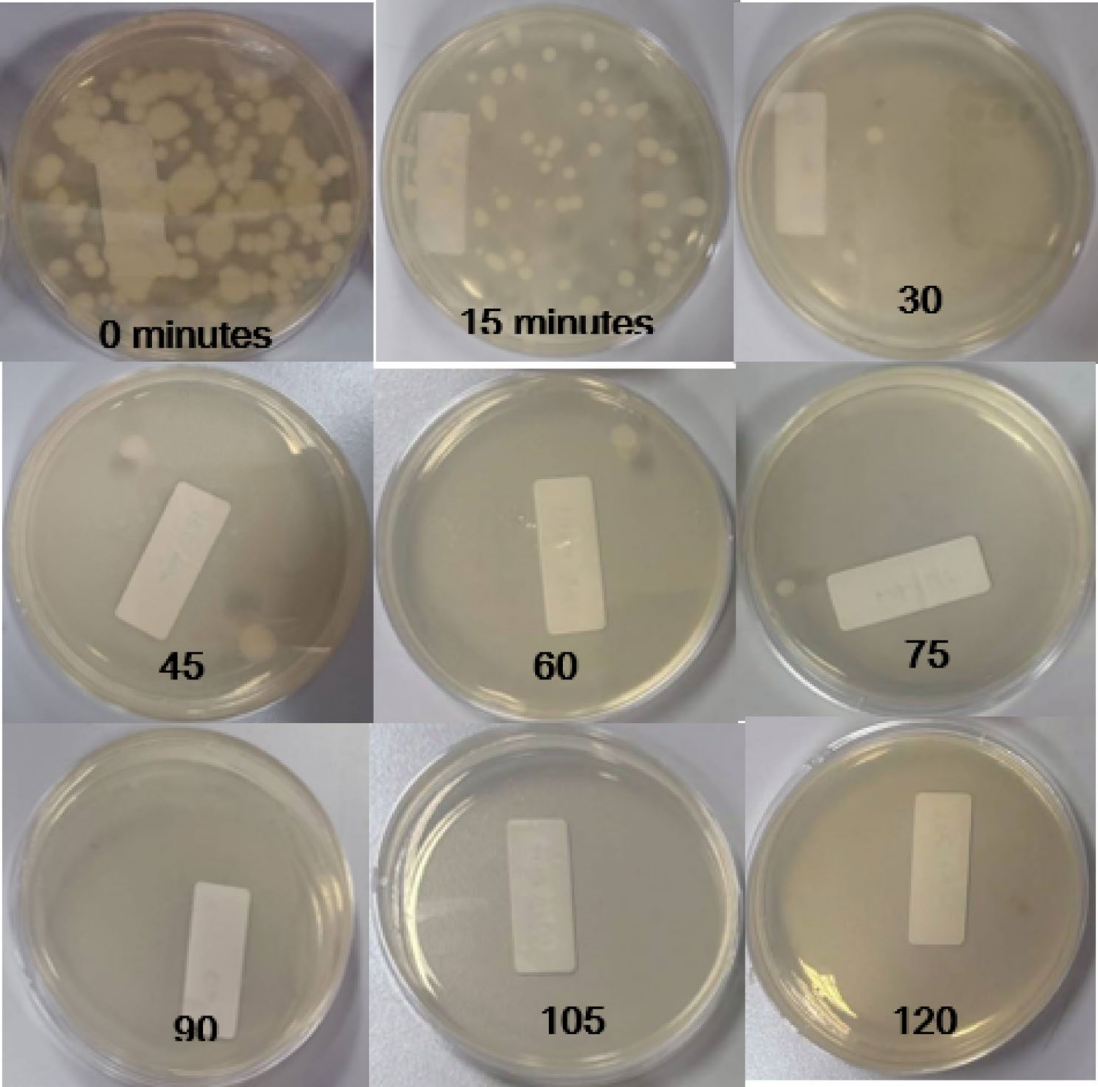
Results of *B. subtilis* Spore Elimination with the UVA + TiO₂ Configuration.

The O_3_ + UVA + TiO_2_ and UVA + TiO_2_ configurations exhibited high and consistent removal efficiencies, demonstrating stable performance in eliminating *B. subtilis* spores. This efficacy is visually demonstrated by the representative Petri dish images for the UVA + TiO_2_ configuration in Fig. 6, where a clear reduction in colonies is observed over time. Both configurations reduced spore concentrations by more than 80% within the first 15 min and achieved near-complete or complete removal within 75 min. In contrast, the O_3_ + UVC + ZnO configuration displayed a slower spore reduction rate compared with the other configurations. While the UVC + ZnO setup achieved rapid initial removal, its performance was unstable over time, with fluctuations in spore concentrations observed during the later stages, suggesting potential microbial persistence or recovery.

### Removal of *B. subtilis* spores on contact surfaces

The removal efficiency of the photocatalytic air purification systems against *B.subtilis* spores on contact surfaces is summarized in Table 2. The UVA + TiO₂ configuration demonstrated the highest overall efficacy among the tested systems. With an initial spore concentration of 10.3 ± 0.6 × 10^4^ CFU/m², the system reduced the load to 1.3 ± 0.6 × 10^4^ CFU/m² (87.10% reduction) within 60 min and further to 0.3 ± 0.6 × 10^4^ CFU/m² at 120 min (96.77% reduction). The O₃+UVA + TiO₂ system showed a similar trend. The initial spore concentration of 10 ± 3 × 10^4^ CFU/m² was reduced to 1.7 ± 2.1 × 10^4^ CFU/m² (83.33% reduction) after 60 min and further declined to 0.67 ± 0.58 × 10^4^ CFU/m² at 120 min (93.33% reduction). In the O₃+UVC + ZnO system, the initial spore concentration was 11 ± 1 × 10^4^ CFU/m². This decreased to 9 ± 1 × 10^4^ CFU/m² (78.79% reduction) after 60 min and further to 2.3 ± 0.6 × 10^4^ CFU/m² at 120 min (87.88% reduction). Finally, the UVC + ZnO configuration showed an initial spore concentration of 11.6 ± 0.6 × 10^4^ CFU/m². A rapid decrease to 0.7 ± 0.6 × 10^4^ CFU/m² (94.12% reduction) was observed at 60 min. Although the surface concentration increased slightly to 1.00 + 1.00 × 10^4^ CFU/m^2^ at 120 min (91.18% reduction) compared with the 60-minute value 0.67 + 0.58 × 10^4^ CFU/m^2^, this fluctuation is well within the measurement uncertainty (approaching + 100%). Therefore, this change is not statistically meaningful and does not indicate regrowth or increased survival. Instead, the large uncertainty observed at later time points primarily reflects the inherent variability of surface sampling in bioaerosol experiments. The UVA + TiO₂ configuration demonstrated the highest removal efficiency, achieving a 96.77% reduction of *B. subtilis* spores within 120 min. This superior performance can be attributed to the photocatalytic oxidation mechanism, in which TiO₂ is activated under UVA irradiation to generate ROS that disrupt microbial cell walls and cellular components^46^. By comparison, the ozone-based systems (O₃+UVA + TiO₂ and O₃+UVC + ZnO) also reduced microbial counts but exhibited lower overall efficiencies than UVA + TiO₂ alone. This reduced performance may be explained by the competitive interactions between ozone and the semiconductor surfaces, where overlapping oxidation pathways hinder the photocatalytic activity and limit the generation of ROS^47^. In the UVC + ZnO configuration, a rapid decline in spore counts was observed within the first 30 min; however, a slight rebound was detected at 120 min, suggesting regrowth or persistence of spores. This phenomenon may be related to the narrower band gap of ZnO, which is well aligned with UVC irradiation^48^, enabling efficient initial photocatalytic activation but with a shorter reactive lifespan compared with TiO₂. Consequently, ZnO-based systems may be less stable for sustained microbial removal.

**Table 2:** Removal of *B. subtilis* spores on contact surfaces (CFU/m²).

Configuration	Contact surfaces (CFU/m²)		
	0 min	60 min	120 min
O_3_ + UVA + TiO_2_	10 ± 3 × 10^4^	1.7 ± 2.1 × 10^4^	0.7 ± 0.6 × 10^4^
UVA + TiO_2_	10.3 ± 0.6 × 10^4^	1.3 ± 0.6 × 10^4^	0.3 ± 0.6 × 10^4^
O_3_ + UVC + ZnO	11 ± 1 × 10^4^	9 ± 1 × 10^4^	2.3 ± 0.6 × 10^4^
UVC + ZnO	11.3 ± 0.6 × 10^4^	0.7 ± 0.6 × 10^4^	1 ± 1 × 10^4^

### Evaluating the efficacy of *B. subtilis* spore removal

Performance comparison of *B. subtilis* spore removal in air

**Fig. 7:**
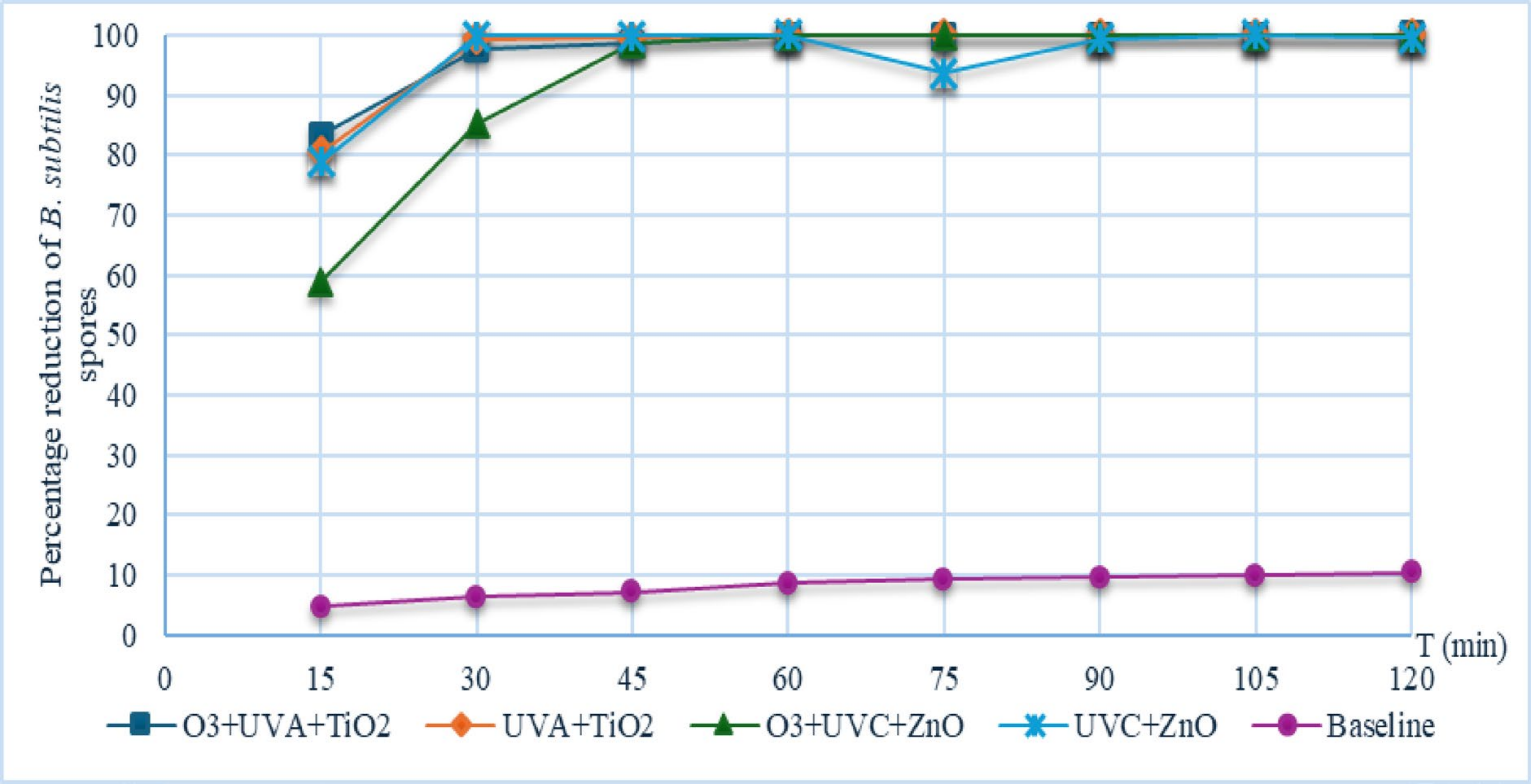
Percentage Reduction of Airborne *B. subtilis* spores throughout the experiment.

As shown in Table 3, at 15 min, most configurations exhibited similar removal efficiencies, with the exception of the O₃+UVC + ZnO system, which reached only 58.91%. From 75 min onward, the UVC + ZnO system showed a decline in efficiency to 93.63%, despite having achieved 100% at earlier intervals. The fluctuations observed throughout the experiment indicate intermittent photocatalytic activity and reduced operational continuity for this specific system. In contrast, the O₃+UVC + ZnO system maintained a stable removal efficiency of 99.96% from 45 to 120 min. The O₃+UVA + TiO₂ system showed stable performance at 99.80% between 60 and 90 min before slightly increasing to 99.97% at 120 min. Among all configurations, the UVA + TiO₂ system exhibited the highest and most consistent removal efficiency, achieving complete removal (100%) from 90 min until the end of the experiment. The overall trends in removal efficiency and the comparative performance of these configurations are visually summarized in Fig. 7. Although the UVC + ZnO system demonstrated high efficacy at certain intervals, its unstable performance reflects the limited durability of ZnO under UVC irradiation^49^. The superior and stable performance of UVA + TiO₂ can be attributed to the moderate energy of UVA, which effectively activates TiO₂ without damaging the catalyst’s coating on the prefilter. In contrast, UVC combined with high concentrations of ozone accelerates the degradation of nano-oxide catalysts. These findings suggest that a photocatalytic system using UVA in combination with TiO₂ is the most suitable technology for *B. subtilis* spore removal in confined environments, such as ambulance cabins, providing both high efficiency and long-term stability.

**Table 3:** Summary of reduction of *B. subtilis* spores with different photocatalytic components.

Configuration	Reduction (%)							
	15	30	45	60	75	90	105	120
O_3_ + UVA + TiO_2_	83.29	97.78	98.48	99.80	99.80	99.80	99.87	99.97
UVA + TiO_2_	80.43	99.41	99.54	99.97	99.97	100	100	100
O_3_ + UVC + ZnO	58.91	85.24	98.67	99.96	99.96	99.96	99.96	99.96
UVC + ZnO	78.76	99.88	99.92	100	93.63	99.30	100	99.61

### Performance comparison of *B. subtilis* spore removal on contact surfaces

At 60 min, the UVC + ZnO configuration demonstrated the highest removal efficiency at 94.12%, followed by UVA + TiO₂ (87.10%), O₃+UVA + TiO₂ (83.33%), and O₃+UVC + ZnO with the lowest efficiency (78.79%). By 120 min, the removal efficiencies of all configurations had increased, with the exception of UVC + ZnO, which decreased from 94.12% at 60 min to 91.18%. At this time point, the UVA + TiO₂ system achieved the highest efficiency at 96.77%, followed by O₃+UVA + TiO₂ (93.33%) while O₃+UVC + ZnO remained the least effective at 87.88%. These results indicate that systems using ultraviolet irradiation combined with a photocatalyst alone provide higher inactivity than those that also incorporate ozone, as summarized in Table 4.

**Table 4:** Summary of reduction of *B. subtilis* spores on contact surfaces with different photocatalytic components.

Configuration	Reduction (%)	
	60	120
O_3_ + UVA + TiO_2_	83.33	93.33
UVA + TiO_2_	87.10	96.77
O_3_ + UVC + ZnO	78.79	87.88
UVC + ZnO	94.12	91.18

### Efficiency of photocatalytic air purifiers in eliminating *B. subtilis* spores in air and on contact surfaces

**Fig. 8:**
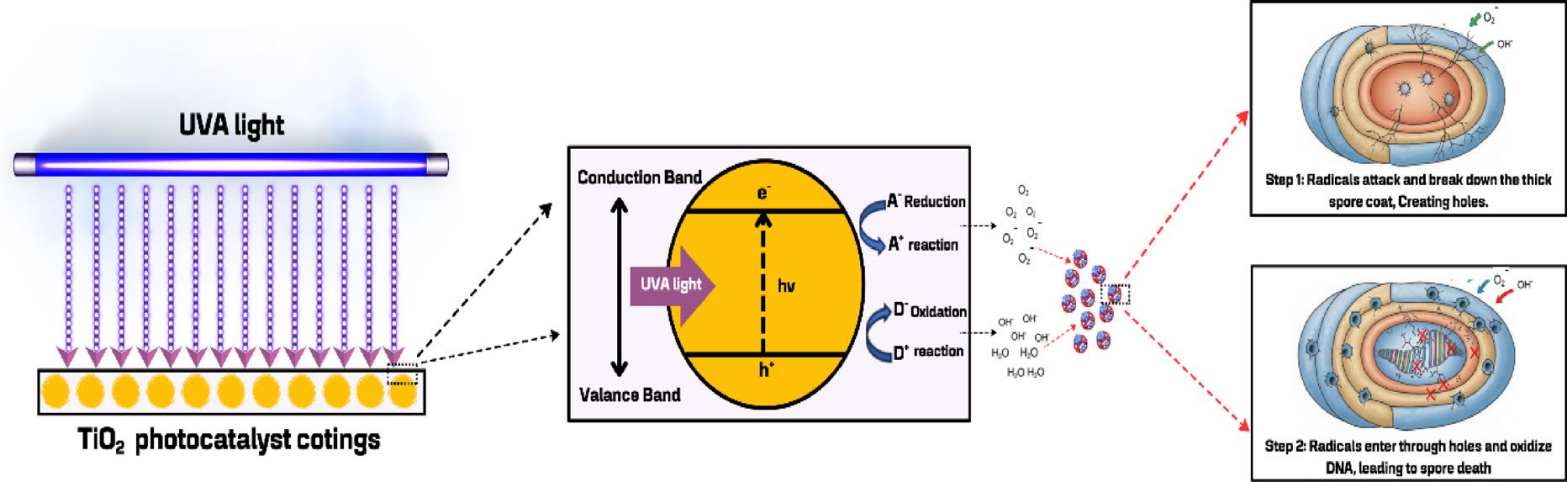
Proposed mechanism of *B. subtilis* spore removal by UVA + TiO₂ photocatalytic system.

The superior performance and stability of the air purifier in removing airborne and surface *B. subtilis* spores is primarily ascribed to the UVA + TiO_2_ configuration. This efficacy is intrinsically linked to the high proportion of the Anatase crystalline phase (more than 70% anatase content, characteristic of the Degussa P25 used)^50^. The Anatase structure confers a band gap energy of 3.20 eV^51^, which is optimally suited for absorbing UVA irradiation at 384 nm. This precise spectral matching promotes a highly balanced and continuous photocatalytic reaction^52^, ensuring the sustained generation of Reactive Oxygen Species (ROS). Furthermore, the inherent high structural stability of the Anatase phase effectively resists photo-corrosion and degradation, thereby mitigating external disturbances from common airborne contaminants and maintaining the robustness of the TiO_2_ coating on the prefilter substrate^53^. However, while the short-term stability is confirmed, the potential for catalyst deactivation of the TiO_2_ surface during prolonged continuous operation, particularly from the accumulation of organic or inorganic residues common in clinical settings, remains a critical concern. Therefore, the long-term stability and resistance to deactivation of the TiO_2_ coating warrants dedicated, long-run investigation to fully validate the system’s operational viability and sustained ROS production for clinical translation. And removal efficiency, which can be explained by the photocatalytic mechanism at the molecular level. Under UVA irradiation, electrons are excited from the valence band of the TiO₂ nanocatalyst coated on the prefilter surface to the conduction band, generating electron-hole pairs (h⁺). These electron-hole pairs lead to the formation of highly oxidative ROS, including hydroxyl radicals (•OH) and superoxide anions (O₂⁻•)^54^, which attack the cell wall of *B. subtilis* spores. *B. subtilis* spores possess a thick and highly resilient multilayered spore coat that normally confers strong environmental resistance^55,56^. However, •OH and O₂⁻• radicals create pores in the sport coat and subsequently penetrate the core, oxidizing the genetic material, ultimately preventing self-repair and leading to spore death^57^, as illustrated in Fig. 8. The high stability of this configuration is further supported by the anatase phase of TiO₂, which exhibits high photosensitivity and an appropriate band gap energy for UVA absorption, enabling long-term and continuous catalytic activity without significant degradation of the catalyst coating on the prefilter surface. This is consistent with the findings of Wei Y. et al. (2023)^58^. In contrast, the UVA + ZnO configuration initially exhibited high spore removal both in air and on surfaces. However, over time, the photocatalytic oxidation process became less continuous, leading to reduced stability and partial recovery of *B. subtilis* spores. This observation aligns with previous studies by Kaur, J. & Singhal, S. (2019) and 60. Zhang et al. (2023)^59,60^, which reported that electron–hole recombination in ZnO limits ROS generation, thereby decreasing sustained photocatalytic activity and allowing spores to survive over extended periods.

Air purification configurations incorporating ozone generators (O₃+UVA + TiO₂ and O₃ +UVC + ZnO) initially showed high removal efficiency. This effect can be attributed to ozone gas penetrating and directly damaging the spore wall of *B. subtilis*. When combined with ROS produced by photocatalysis, the antimicrobial activity was further enhanced. Over time, however, the accumulation of ozone at elevated concentrations accelerated the degradation of the catalyst coating on the prefilter surface. This degradation was more pronounced under UVC irradiation, where the combined action of ozone and high-energy photons hastened photocatalyst deterioration and reduced long-term disinfection efficiency. This was reflected in the O₃+UVC + ZnO system, which exhibited the lowest surface removal efficiency after 120 min. Mechanistically, the decline may stem from reduced generation of ROS such as •OH and O₂⁻•, which normally oxidize key biomolecules within spores including proteins, lipids, and nucleic acids. Importantly, ozone is also a respiratory toxicant^61^, therefore, despite its short-term efficacy, ozone-based systems may not be suitable for deployment in ambulances, where they pose health risks to medical staff, patients, and accompanying relatives.

Given the absence of prior investigations on air purification in ambulances, our study compared the tested system with air purifiers previously evaluated in hospital environments, in which high patient density creates significant risks of airborne transmission. Consistent with the findings of Yu, L. et al. (2025)^62^ and Xiong et al. (2022)^25^, our results demonstrate that the UVA + TiO₂ photocatalytic system provides highly efficient removal of *B. subtilis* spores and, by extension, other pathogenic microorganisms. Importantly, this system exhibited sustained performance throughout the experimental period, indicating long-term stability of the photocatalytic oxidation process. Unlike ozone-based configurations, it does not generate harmful secondary pollutants, thereby ensuring safe operation in the confined environment of ambulance cabins. Therefore, the integration of UVA + TiO₂ air purification systems into emergency medical services offers a promising and practical approach for infection control, combining high microbial removal efficiency with safety for medical personnel, patients, and accompanying relatives.

The simulated test chamber was specifically designed to replicate the interior volume of an ambulance patient compartment (8.998 m^3^). To facilitate a controlled and standardized comparison of the four photocatalytic configurations, the air purification units were centrally positioned. While this central location corresponds to the actual patient area in a real ambulance, its primary purpose here was to ensure a uniform assessment of disinfection performance under controlled experimental conditions. Consequently, the findings of this study primarily reflect the intrinsic efficiency of each configuration under controlled airflow and exposure conditions, with the UVA + TiO_2_ system demonstrating the highest spore removal efficiency. However, the translation of these results to real-world ambulance environments necessitates considering several crucial factors. These include the complex airflow dynamics within the cabin, identifying appropriate installation locations to maximize the removal of aerosol particles in the 1–100 μm range (as highlighted by the reviewer), and maintaining sufficient air exchange rates to ensure optimal operational performance (e.g., > 12 ACH, as recommended by agencies such as ASHRAE or CDC for controlled healthcare settings)^63^. These parameters will be critical for the effective deployment of photocatalytic air purification systems in future ambulance applications.

The reduction in surface contamination observed in this study is attributable primarily to decreased airborne spore concentration, which subsequently reduces particle deposition onto surrounding surfaces. Because photocatalytically generated ROS are extremely short-lived and have minimal diffusion distances, they are unlikely to travel beyond the catalyst surface or contribute directly to inactivation on distal surfaces. Therefore, the surface reduction effect in this study reflects an indirect benefit of improved airborne decontamination rather than direct ROS-mediated surface disinfection. Unlike HEPA filtration, PCO additionally degrades captured biological material on the catalyst surface, potentially reducing long-term maintenance requirements. However, direct comparison with HEPA was beyond the scope of this study and should be addressed in future work.

## Limitations and scope

The presented results reflect the efficacy of *B. subtilis* spore reduction, achieved under fixed conditions: UVA and UVC light intensity at 6 W, an initial spore concentration of 1.5 10^8^ CFU/ mL, and a prefilter area of 20 × 20 cm. These specific parameters were determined to ensure the air purifier system remains compact for future use in ambulances, thereby avoiding obstruction of critical medical tools and equipment. Therefore, the results presented reflect the maximum potential spore reduction achieved only under these specified and practical conditions. Future research should focus on in-depth studies to determine the optimal PCO parameters necessary for maximizing efficiency before use in ambulance settings.

## Conclusion

This study demonstrates that the UVA + TiO₂ configuration is the most effective and stable system for removing *B. subtilis* spores in both the air and on surfaces. It achieved complete airborne spore removal within 75 min and reduced surface contamination by up to 96.77%. These results highlight the system’s suitability for integration into ambulance air purification units, where the confined environment and continuous operation demand both high efficiency and safety. While the study was conducted under controlled experimental conditions, further investigations in real ambulance settings are warranted to validate its performance against clinically relevant multidrug-resistant organisms. Overall, the application of UVA + TiO₂ photocatalysis offers a promising and safe approach to strengthening infection control in emergency medical service vehicles.

## Data Availability

The data that support the findings of this study are available from the article or from the corresponding authors upon reasonable request.
